# Does strontium coated titanium implants enhance the osseointegration in animal models under osteoporotic condition? A systematic review and meta-analysis

**DOI:** 10.1038/s41405-024-00220-9

**Published:** 2024-08-24

**Authors:** Osama Sayed, Mohamed Mahmoud Abdalla, Ayman Elsayed, Yehia El-Mahallawy, Haytham Al-Mahalawy

**Affiliations:** 1 Oral and Maxillofacial Surgery Department, Faculty of Dentistry, Fayoum University, Fayoum, Egypt; 2 Paediatric Dentistry, Faculty of Dentistry, The University of Hong Kong, Hong Kong, China; 3 Dental Biomaterials, Faculty of Dental Medicine Al-Azhar University, Cairo, Egypt; 4 Faculty of Medicine, Fayoum University, Fayoum, Egypt; 5 Assistant Professor, Oral and Maxillofacial Surgery Department, Faculty of Dentistry, Alexandria University, Alexandria, Egypt; 6 Professor and head of the Oral and Maxillofacial Surgery Department, Faculty of Dentistry, Fayoum University, Fayoum, Egypt

**Keywords:** Dental materials, Fixed prosthodontics

## Abstract

**Purpose:**

The aim of this study was to systematically review the literature to address the effect of strontium modified titanium implants on the osseointegration in the presence of osteoporotic conditions through animal models.

**Materials and methods:**

The databases (PubMed, Scopus, Web of Science, and EBSCO) were searched electronically, and manual searches were performed till December 2022 to identify preclinical studies on the osseointegration of strontium coated titanium implants in animals with induced osteoporotic conditions. The primary outcomes were the bone-implant contact percentage (BIC%), bone area (BA) from the histomorphometric analysis, and the osseointegration parameters from biomechanical tests; the secondary outcomes were the osseointegration parameters from the micro computed tomography.

**Results:**

Nineteen articles were included for the quantitative analysis on basis of the inclusion criteria. The results revealed that Sr-modified implants showed a significant 19.05% increase in BIC, and 15.01% increase in BA. The results of biomechanical tests indicated a significant effect in favor of Sr-coated implants. Furthermore, Results of the secondary outcomes supported the significant advantages of Sr-coated implants over the un-coated implants. The overall, systematic analysis of implants osteointegration parameters proved a significant increase in favor of Sr-coated titanium implants (*P* < 0.01).

**Conclusion:**

The present results provide evidence that strontium-coated titanium implants enhanced the osseointegration in animal models under osteoporotic condition as this surface modification techniques have improved the mechanical and biological properties of the titanium implants.

## Introduction

Dental implants have become the most effective and predictable means of teeth replacement for both partially and totally edentulous patients due to their high success and survival rates [1]. This success depends on achieving and maintaining a direct structural and functional connection between ordered living bone and the surface of load carrying implant in a process known as osseointegration. According to Albrektsson et al., Implant material biocompatibility, implant surface, implant design, surgical and loading techniques, and host tissue condition are the main six factors that influence osseointegration [2].

In spite of all advancements in the field of dental implantology, there still exist a significant population of patients where dental implant treatment is considered as a relative contraindication due to the unpredictable outcomes where there is no consensus in the literature regarding the success and survival rates of dental implants in these patients. These relative contraindications include any medical conditions that affect bone metabolism or the patient’s ability to heal, such as diabetes, osteoporosis, immune compromise conditions, and medical treatments such as chemotherapy [3–5].

Osteoporosis is a generalized skeletal disorder characterized by reduction in bone density and deterioration of the microarchitecture of the bone tissue due to high bone turnover rates and imbalance in bone remodeling, where bone resorption exceeds bone formation, leading to bone fragility with greater risk of bone fracture and decreased capacity of bone to repair [6, 7]. Several previous experimental studies have reported the negative effect of osteoporosis on the extraction socket healing [7], as well as on the osseointegration of dental implants [8–10].

The aging population of humans are progressively increasing, so more people will be affected by osteoporosis and missing teeth, so it is expected that the number of osteoporotic patients who are in need to replace their missing teeth by dental implants will increase all over the world [11]. Thus, it is essential to develop a scientifically validated technologies to improve implant osseointegration in such conditions to achieve more predictable outcomes.

Currently, the majority of implant systems available in the market are using the well-established sandblasting acid etching (SLA) technique as a standard surface treatment modality to greatly improve the roughness, hydrophilicity, cell adhesion, and consequently the bone-implant integration [12]. Nevertheless, in some challenging bone situations, such as low bone quality and quantity, the SLA surface lacks sufficient pro-osteogenic bioactivity to induce adequate osteogenesis to ensure successful implant osseointegration [13]. Therefore, to promote the mechanical and biological performance of titanium implants, a surface modification with co-osteogenic properties is highly recommended to enhance the osseointegration and achieve a more predictable outcomes in such challenging bone conditions.

Strontium (Sr) is an essential microelement in the human body that, like calcium, has powerful bone-seeking characteristics. It is currently used in the treatment of osteoporosis [14]. In contrast to the anticatabolic drugs like bisphosphonates that decrease bone resorption, and the anabolic dugs like parathyroid hormone that enhance bone deposition, Sr has a unique dual mechanism of action as it is simultaneously induce bone formation through increasing the osteoblastic activities and at the same time prevent bone resorption through reduction of osteoclastic activities. This dual action has been reported in several previous in vitro experiments [15, 16]. Furthermore, several previous in vivo studies, performed in both normal and osteoporotic animal models, showed that Sr-coated titanium implants have significantly better osseointegration parameters than Sr-free implants [17–19].

Two previous systematic reviews have proved the positive effect of Sr-coated titanium implants on the osseointegration in healthy, non-osteoporotic conditions [20, 21]. Furthermore, a prior systematic review has demonstrated the beneficial effect of strontium supplementation on implant osseointegration in osteoporotic settings [22]. However, the efficacy of Sr coated titanium implants on improving new bone formation and enhancing implant osseointegration in the presence of osteoporotic conditions is still unclear. Therefore, the aim of this study was to systematically review the effect of Sr modified titanium implants on the osseointegration in the presence of osteoporotic conditions through animal models.

## Methods

This systematic review and meta-analysis follow the Preferred Reporting Items for Systematic Reviews and Meta-Analyses (PRISMA) standards and adheres to the guidelines from the Cochrane Handbook [23, 24].

### PICO question

To answer the research question: “Does strontium coated titanium implants enhance the osseointegration in animal models under osteoporotic condition?”, the Population, Intervention, Control, and Outcomes (PICO) approach was applied (Table 1). The Population was animals with induced osteoporotic conditions received endosseous implants; the Intervention was Strontium-coated titanium implants; the Comparator was titanium implants without strontium coating; the primary outcomes were the bone-implant contact percentage (BIC%) and the bone area (BA) mainly from the histomorphometric analysis, and the osseointegration parameters from biomechanical tests; the secondary outcomes were the osseointegration parameters from the micro computed tomography.

**Table 1:** PICO.

PICO	
Question	Does strontium-coated titanium implant enhance the osseointegration in animal models with osteoporotic conditions?
Participants	Osteoporotic animal models receiving endosseous implants.
Intervention	Titanium endosseous implants coated with strontium.
Comparison	Titanium endosseous implants without strontium coating.
Primary Outcomes	*Histomorphometric analysis*: Bone-implant contact percentage BIC% Bone area BA *Biomechanical tests*: Removal torque (N.cm) Max. pull out force (N) Max. push out force (N) Push-in force (N)
Secondary outcomes	*Micro-CT osseointegration parameters*: Bone volume/Total volume Bv/Tv. Trabecular bone Thickness Tb.Th (micro m) Trabecular Separation Tb.Sp (micro m) Trabecular number Tb.N (1/mm) Connectivity Density Conn.D

### Eligibility criteria

The inclusion criteria that were applied were as follows: [1] in-vivo models with induced osteoporosis; [2] preclinical studies that provide any quantitative data for the primary or secondary outcomes mentioned above.

The following exclusion criteria were applied: [1] All secondary works, such as meta-analyses and reviews; [2] in vitro studies; [3] studies that address the hybrid effect of Sr and other elements; [4] non-osteoporotic animal studies; [5] studies with systemic administration of Osseo-inductive drugs.

### Search strategy and study selection

A comprehensive search was conducted, including the databases MEDLINE via PubMed, Web of Science, Scopus, and EBSCO, until December 2022 for articles that matched the inclusion criteria without language restrictions. The following keywords were used as search terms in PubMed: [(“strontium” OR “Sr” OR “Strontium[Mesh] OR Strontium-incorporated OR Strontium-surface OR strontium-coat OR strontium-coating OR strontium-oxide OR Sr-coat OR Sr-HA OR NT-Sr OR nano-Sr OR nano-strontium OR strontium-substituted OR strontium-functionalized OR strontium-loaded OR Strontium-modified OR Sr-modification OR SLA-Sr) AND (titanium OR Ti OR implant OR implantation OR implants OR SLA OR “Titanium[Mesh] OR “Dental Implants[Mesh]) AND (“osteoporosis OR osteoporotic OR osteopenia OR osteopenic)]. (The comprehensive search strategies for all databases are provided in supplementary data).

The included articles were screened in three steps. The first step implied importing the results from electronic databases to a Microsoft Excel sheet using EndNote Software (*EndNote 20, Clarivate: Philadelphia, PA*). The second step was done by two independent authors and included title and abstract screening of the articles imported to the Excel sheet. The third step was the full-text screening of the included citations from step 2. Additionally, the references of the included articles were manually searched for possible missed studies.

### Data collection

Two independent reviewers separately collected three categories of data from each included study: the first category is the baseline and characteristics of the included subjects, such as the author, year, sample size, animal model, sex, age, Sr integration methods onto the implant coating, Sr concentration, implant size and design, the implantation site and follow-up periods. The second category included the outcomes of analysis, mainly: BIC, BA and biomechanical tests. Plot digitizer 2.6.9 (https://plotdigitizer.com) was used to compute the quantitative values expressed only in graphs. The third category included data for risk of bias assessment. The process of data collection was done using Microsoft Excel.

### Risk of bias assessment and quality of reports

The risk of bias tool provided by SYRCLE (Systematic Review Centre for Laboratory Animal Experimentation) [25] was used to assess the reported methodology of the included studies. Two authors assessed the risk of bias among included studies. The tool assesses proper randomization, allocation concealment, adequate blinding, and outcome reporting through nine domains. Each domain is put to either “low”, “unclear”, or “high” risk of bias. For the quality of reports, the modified ARRIVE guidelines (Animal Research: Reporting of In Vivo Experiments) [26] were applied, Two reviewers assigned a score of 0 (not reported) or 1 (reported) to each item out of 23 items, then the overall score of each study was evaluated.

### Analysis

The meta-analysis of this study was performed using Review Manager Software (*RevMan 2020, The Cochrane Collaboration*). The study included continuous outcomes which were analyzed using mean difference (MD) for BIC, BA, bone architecture parameters from the Micro-CT, and standard mean difference (SMD) for biomechanical tests with a 95% confidence interval (CI). A random-effect model was used due to the substantial heterogeneity. To measure the presence of inconsistency among the studies, the I2 and the *p*-value of the Chi-square tests [24] were used. Values of *P* < 0.1 or I2 > 50% were significant indicators of the presence of heterogeneity. Sensitivity analysis was used in a trial to solve the inconsistency of heterogeneous outcomes. Finally, Forest plots to visualize the estimated effect sizes and funnel plots to identify publication bias were generated if 10 or more studies were included.

## Results

### Results of literature search

**Fig. 1:**
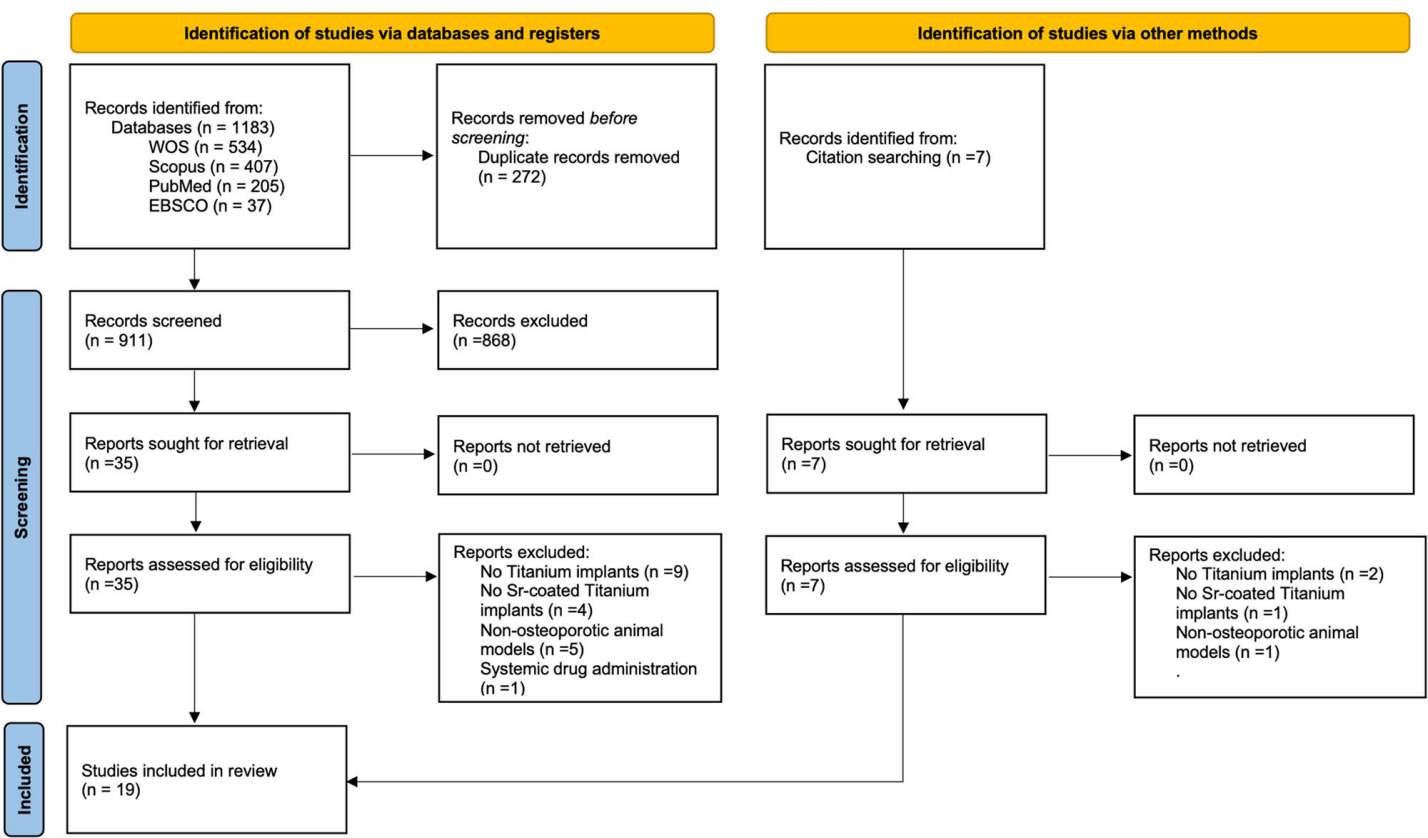
PRISMA Flowchart for the process of study selection.

The study screening process is summarized in Fig. 1. The electronic search yielded a total of 1183 records while the manual search identified 7 records of relevance. After duplicate removal, a subsequent title and abstract screening of the 911 articles concluded that 42 articles were eligible for inclusion. The full texts were then assessed, and 19 articles were included for the qualitative and quantitative synthesis on basis of the inclusion criteria. (Full-text excluded studies are presented in the supplementary data).

### Characteristics of included studies

Table 2, summarizes the general characteristics of the included studies. In summary, nineteen studies were included in the systematic review to investigate the effect of strontium surface coating on implant osseointegration in animal with induced osteoporosis [17, 27–44]. The majority of investigators used rodents, as fifteen research utilized rats, three studies used rabbits, and one study used sheep. Eleven studies used the femur for implantation, six studies used the tibia, one study used both the tibia and the femur, and one study used the mandibular angle. The overall follow-up periods ranged from 2 to 12 weeks.

**Table 2:** Characteristics of the included studies.

Study	Subject model, sex & age	Intervention groups	Follow-up periods	Sr integration methods	Assessment methods	Outcomes
Geng [27]	Female New Zealand white rabbits,1 Y	Ti, Ti-Ca3.7, Ti-Ca4.9, Ti-Sr3.7 and Ti-Sr4.9 (The numbers indicate pH value of preparation)	1, 2, 3 m	Electrochemical deposition	micro-CT, Histological analysis, biomechanical push-in test.	Tb⋅Th, (BIC), push-in force.
Katunar [28]	Female Sprague Dawley rats	Ti implants, Ti BG, and Ti BGSr	15 & 30 d	Sol-gel technique	Micro-Raman spectroscopy maps and histomorphometric analysis.	BA, Tb. Th.
Li [30]	Female Sprague Dawley rats,6 m	Hybrid micro/nanorough titanium strontium-loaded (MNT-Sr) surface, smooth titanium surface (ST), microrough titanium surface (MT) and strontium-loaded nano titanium NT-Sr	12 w	Magnetron sputtering	Micro-CT scanning, Histological analysis, pull-out test	Tb.Th,Tb.N, Tb.Sp, BV/TV, BIC, maximum pull-out force
Li [29]	Female Sprague Dawley rats, 3 m	HA-coated implants and 10% SrHA coated implants	12 w	Sol-gel technique	Histomorphometry, micro-CT evaluation and the maximal push-out force	BIC, BA, BV/TV, Tb.Th,Tb.N, Tb.Sp, Conn.D, and maximal push-out force
Li [30]	Female Sprague Dawley rats, 3 m	HA-coated implants and 10% SrHA coated implants	8 w	Chemical coprecipitation	Micro-CT assessment and Histomorphometry;	BIC, BV/TV, Tb.N,Tb.Th, Conn.D and Tb.Sp
Liang [31]	Female Sprague Dawley rats, 3 m	Implants with a Sr coating and implants without an Sr coating	4 w	Electrochemical deposition	Histomorphometry, Fluorescent histology. Biomechanical testing	BIC, BV/TV, removal torque
Lin [32]	Female New Zealand rabbits, 6 m	SHAM‐SLA‐Sr, OVX‐SLA‐Sr, SHAM‐SLA and OVX‐SLA.	3 & 6 w	Hydrothermal treatment	Removal torque test and histomorphometric analysis	removal torque values, BIC%, BA%
Liu [33]	Female sheeps 4 ± 0.7 y	Hybrid micro/nanorough titanium strontium-loaded (MNT-Sr) surface, smooth titanium surface (ST), microrough titanium surface (MT) and strontium-loaded nano titanium NT-Sr	12 w	Magnetron sputtering	Micro-CT, Histomorphometry, Pull out test;	BV/TV, Tb.Sp, Tb.Th. BIC, the maximum pull-out force
Mi [34]	Female Sprague Dawley rats, 10 weeks	Sham, OVX, OVX + Ti, OVX + TiO2-NTs, OVX + NT-Sr1h, and OVX + NT-Sr3h	8 w	Hydrothermal treatment	Micro-CT, Histological analysis and Serum analysis.	BV/TV, Tb.Sp, Tb.Th, Tb.N.
Offermanns [35]	Female Wistar rats	Unmodified Ti implants; Ti-Sr-O (22 minites-wash); Ti-Sr-O (no wash); Ti-Sr-O (industrial wash).	6 & 12 w	Magnetron sputtering	Histomorphometry.	BIC, BA
Shen [36]	Female Sprague Dawley rats	Normal [Sr25% (*n* = 5) & Sr1000% (*n* = 5)] and osteoporotic groups [Ti (*n* = 10), Sr0% (*n* = 10), Sr25% (*n* = 10) & Sr100% (*n* = 10)].	6 &7 d, 1 m	Micro-arc oxidation (MAO)	Micro-CT, Histomorphometry, Oxidative stress level and Macrophage polarization	BV/TV,Tb.N, Tb.Th, Tb.Sp
Tao [37]	Female Sprague Dawley rats, 3 m	HA, 5% Sr-HA, 10% Sr-HA, and 20% Sr-HA	12 w	Electrochemical deposition	Histological, micro-CT, and pushout tests	BV/TV, Tb.Th, Tb.N,Tb.Sp,Conn.D. Max. push out force, BIC and BA
Tao [38]	Female Sprague Dawley rats, 3 m	HA, 10% Zn-HA,10% Mg-HA and 10% Sr-HA	12 w	Electrochemical deposition	Histological, micro-CT, and pushout tests	BV/TV, Tb.Th, Tb.N,Tb.Sp,Conn.D. Max. push out force, BIC and BA
Tao [39]	Female Sprague Dawley rats, 3 m	HA,10% Sr-HA group, PTH group and PTH+Sr group	12 w	Electrochemical deposition	Histological, micro-CT, and push-out tests.	BV/TV, Tb.Th, Tb.N,Tb.Sp,Conn.D. Max. push out force, BIC and BA
Wang [40]	Female Sprague Dawley rats	AH-Ti and AH-Ti/Sr90 (deposition durations 90 min)	4 w	Magnetron sputtering	Micro-CT and histological analysis.	Tb.N, BV/TV, Tb.Sp, Tb.Th, Conn.D.
Wen [41]	Female Sprague-Dawley rats, 3 m	(i) Ti group (*n* = 8); (ii) TiO2 group (*n* = 8); (iii) AT–TiO2 group (*n* = 8); (iv) Sr–TiO2 group (*n* = 8).	8 w	Micro-arc oxidation	Sequential fluorescent labeling and Histomorphometric analysis	BIC
Zhang [42]	Female Sprague Dawley rats, 3 m	HA, 2.5% Zn-HA, 2.5% Mg-HA, and 2.5% Sr-HA	4, 8, 12 w	Electrochemical deposition	Histomorphometric Analysis	BIC, BA
Zhao [43]	Female New Zealand rabbits, 5–6 m	A: machined surface, B: MAO and C: MAO-Sr-simvastatin	4, 8, 12 w	Micro-arc oxidation (MAO)	Pull-Out Tests, Histomorphometric analysis	Maximum pull-out force, BIC
Zhu [44]	Female Sprague Dawley rats, 12 weeks	Sham+Ti, Ti, TiO2, TiO2+Sr and TiO2+Sr+ICA.	4 w	Hydrothermal treatment	Micro-CT assessment and Histomorphometry.	BV/TV, BIC

*Sr* strontium, *HA* hydroxyapatite, *Ti* titanium, *SLA* sandblasted acid-etched, *Micro-CT* microcomputed tomography, *BIC* bone to implant contact, *BA* bone area, *MAO* micro-arc oxidation, *RTT* removal torque test, *BV/TV* bone volume/total volume, *Tb.Sp* trabecular spacing, *Tb.N* trabecular number, *Tb.Th* trabecular thickness, *Conn.D* the mean connective density.

The implant characteristics in the included studies were as follows; a total of 506 implants were used. In terms of implant design; 9 studies used rod-shaped implants, 9 studies utilized screw-shaped implants, and one study used wire-shaped implants, the implant diameter ranged from 0.8 to 4.3 mm and the implant length ranged from 4 to 20 mm.

### Results of risk of bias and quality of reports assessment

**Fig. 2:**
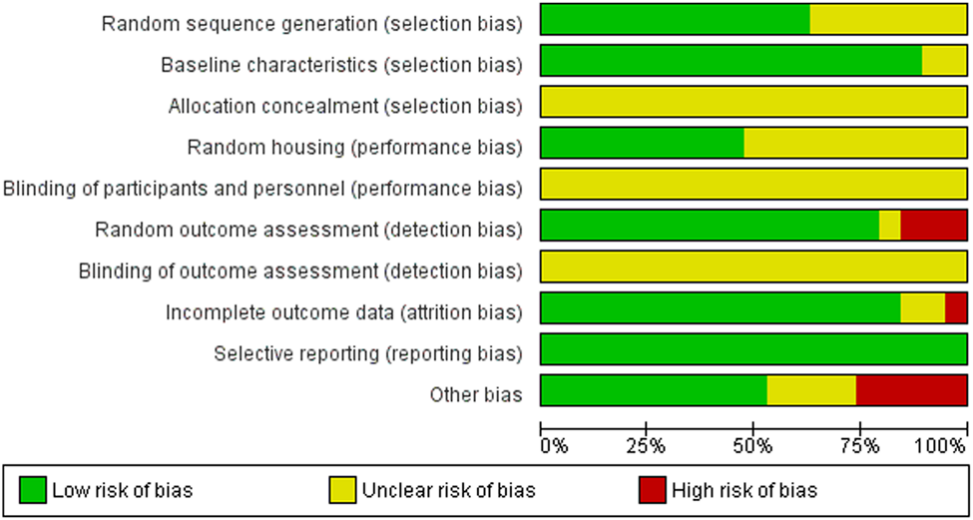
Risk of bias (RoB) evaluation by the Systematic Review Centre for Laboratory animal. Experimentation (SYRCLE) RoB assessment tool.

SYRCLE risk of bias assessment tool results are shown in Fig. 2. The ARRIVE criteria of the included studies recorded an average score across the board of 19.15(±1.46) out of a maximum of 23, All the included studies reported correctly on the title, abstract, introduction, ethical statement, species, surgical procedure, outcomes assessment, and statistical analysis. On the other hand, none of the included studies reported 3Rs or the presence of adverse events (Table 3).

**Table 3:** ARRIVE criteria reported by the included studies.

ARRIVE	Geng [27]	Katunar [28]	Li [30]	Li [29]	Li [30]	Liang [31]	Lin [32]	Liu [33]	Mi [34]	Offermanns [35]	Shen [36]	Tao [37]	Tao [38]	Tao [39]	Wang [40]	Wen [41]	Zhang [42]	Zhao [43]	Zhu [44]
1.Title	1	1	1	1	1	1	1	1	1	1	1	1	1	1	1	1	1	1	1
Abstract																			
2. Species	0	1	1	1	1	1	1	1	1	1	0	1	1	1	0	1	1	1	1
3. Key finding	1	1	1	1	1	1	1	1	1	1	1	1	1	1	1	1	1	1	1
Introduction																			
4. Background	1	1	1	1	1	1	1	1	1	1	1	1	1	1	1	1	1	1	1
5. Reasons for animal models	0	1	0	1	1	1	1	1	1	1	0	1	1	1	0	1	1	1	1
6. Objectives	1	1	1	1	1	1	1	1	1	1	1	1	1	1	1	1	1	1	1
Methods																			
7. Ethical statement	1	1	1	1	1	1	1	1	1	1	1	1	1	1	1	1	1	1	1
8. Study design	1	1	1	1	1	1	1	1	1	1	1	1	1	1	1	1	1	1	1
9. Experimental procedures	1	1	1	1	1	1	1	1	1	1	1	1	1	1	1	1	1	1	1
10. Experimental animals	1	1	1	1	1	1	1	1	1	1	1	1	1	1	1	1	1	1	1
11. Accommodation and handling of animals	0	0	0	1	1	0	1	0	1	1	0	1	1	1	1	0	0	0	1
12. Sample size	0	1	1	1	1	1	1	1	1	1	1	1	1	1	1	1	1	1	1
13. Assignment of animals to experimental groups	1	1	1	1	1	1	1	1	1	1	1	1	1	1	1	1	1	1	1
14. Anaesthesia	1	1	1	0	0	1	1	1	1	1	0	1	0	0	0	0	1	1	1
15. Statistical methods	1	1	1	1	1	1	1	1	1	1	1	1	1	1	1	1	1	1	1
Results																			
16. Experimental results	1	1	1	1	1	1	1	1	1	1	1	1	1	1	1	1	1	1	1
17. Results and estimation	1	1	1	1	1	1	1	1	1	1	1	1	1	1	1	1	1	1	1
**Discussion**																			
18. Interpretation and scientific implications	1	1	1	1	1	1	1	1	1	1	1	1	1	1	1	1	1	1	1
19. 3Rs reported	0	0	0	0	0	0	0	0	0	0	0	0	0	0	0	0	0	0	0
20. Adverse events	0	0	0	0	0	0	0	0	0	0	0	0	0	0	0	0	0	0	0
21. Study limitations	0	1	1	1	1	0	1	1	0	1	1	1	1	1	0	1	1	0	0
22. Generalization/ applicability	1	1	0	1	1	1	1	1	1	1	1	0	0	1	1	1	1	0	1
23. Funding	1	1	1	1	1	1	1	1	1	1	1	1	1	1	1	1	1	0	1
Total score	16	20	18	20	20	19	21	20	20	21	17	20	19	20	17	19	20	17	20

### Analysis of outcomes

To assess the implant osseointegration in test subjects with osteoporosis the included studies used histological, radiographical, as well as biomechanical analysis. Histological and radiographical analysis were used mainly to evaluate the bone implant contact (BIC), bone area (BA), and bone microstructure parameters: Bone volume / Total volume BV/TV, Trabecular Thickness Tb.Th (micro m), Trabecular Separation Tb.Sp (micro m), Trabecular number Tb.N (1/mm), Connectivity Density Conn.D. Moreover, different biomechanical tests were used including removal torque test, maximum pull out test, maximum push out test and maximum push-in test. The overall, systematic analysis of implants osseointegration parameters proved a significant increase in favor of Sr-coated titanium implants.

### Histomorphometric parameters assessment

**Fig. 3:**
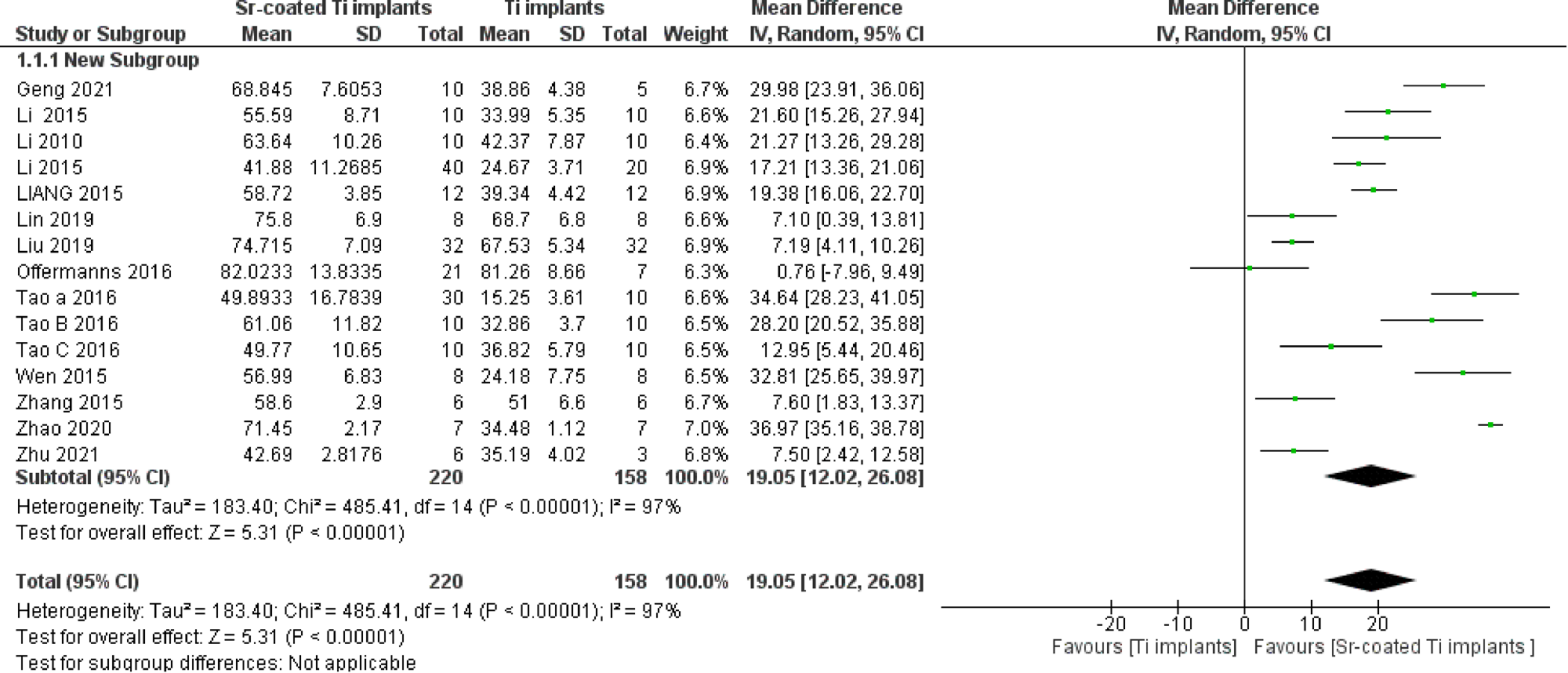
Bone to implant contact (BIC) forest plot.

**Fig. 4:**
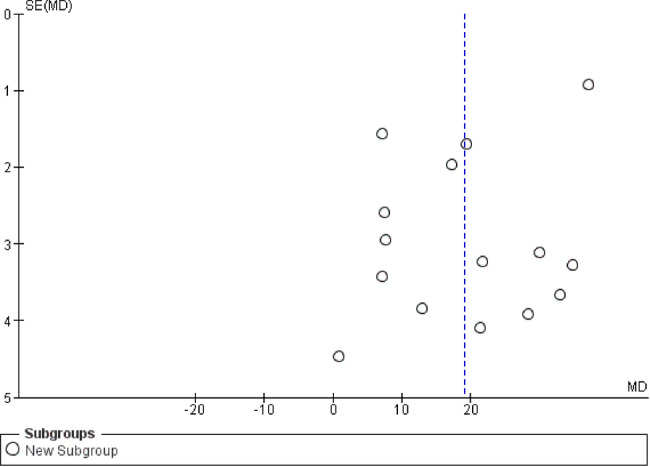
Bone to implant contact (BIC) funnel plot.

Regarding the primary outcomes, the BIC pooled analysis of the 15 included studies showed a statistically significant superior results in favor of Sr coated implants with 19.05% increase (*P* < 0.00001) despite considerable heterogeneity (Chi² = 485.41, (*P* < 0.00001), I² = 97%) (Fig. 3). Subgroup analysis according to the animal model, implantation location, and follow-up period could not explain the source of heterogeneity neither did the sensitivity analysis. Furthermore, the funnel plot illustrated the presence of publication bias (Fig. 4).

**Fig. 5:**
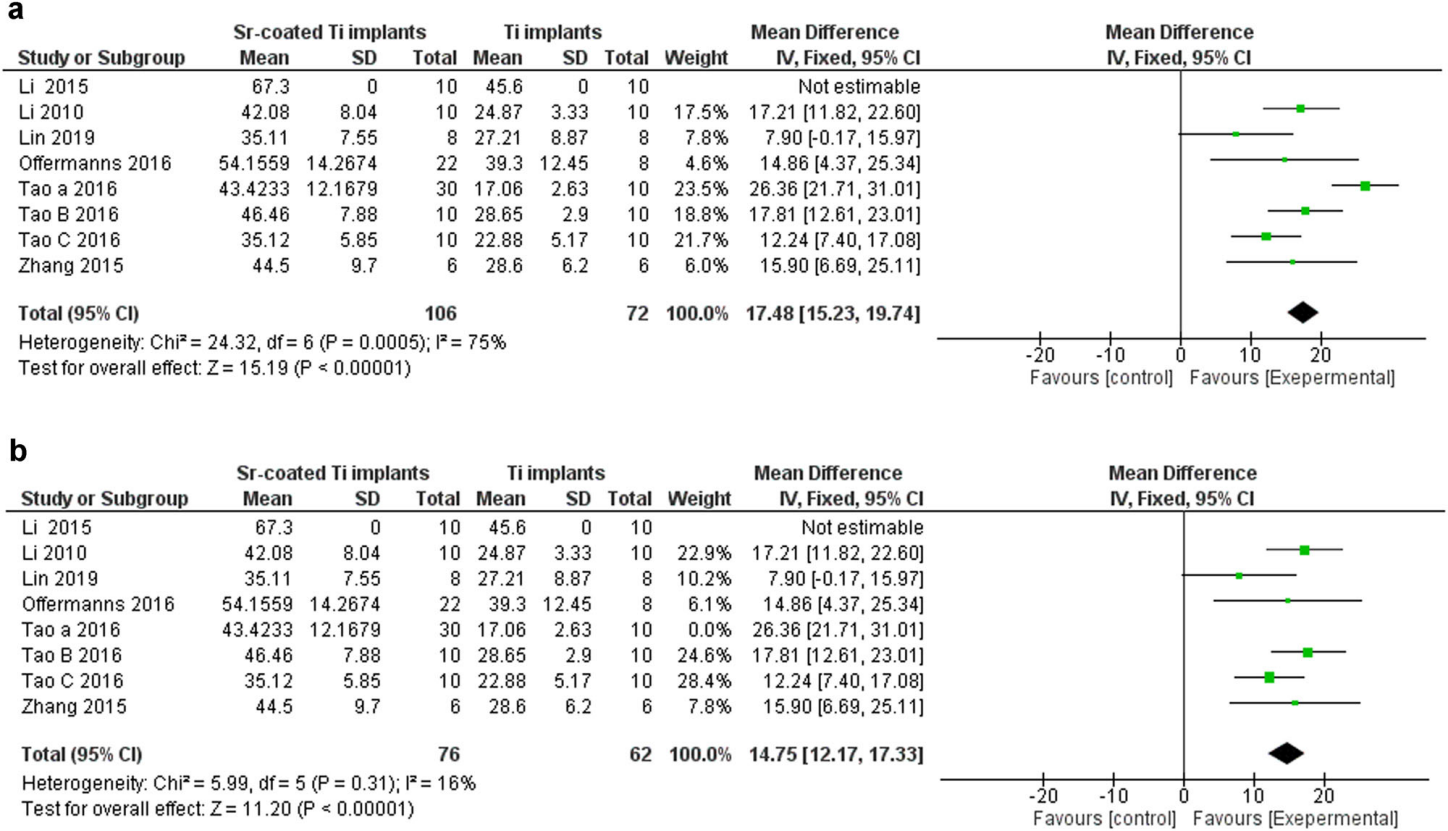
Bone area (BA) forest plot senstivity analysis. **a** BA forest plot. **b** BA forest plot after excluding the sources of heterogenicity and performing sub-group analysis.

The Bone Area (BA) *n* = 9 studies significantly increased in Sr-coated group (15.01%, *P* < 0.00001) with considerable heterogeneity (I² = 75%), the sensitivity test by excluding one study resulted in homogenous results in favor of Sr-coated implants (*P* = 0.31); I² = 16%) (Fig. 5).

Results of the secondary outcomes supported the advantages of Sr-coated implants, Bone volume per total volume (BV/TV) (*n* = 11 studies) was significantly higher in the Sr-coated group (MD = 12.48%, *P* < 0.00001); likewise, connectivity density (Conn.D) (*n* = 6 studies) was also better in the Sr-coated group (MD = −15.16 mm^−3^, *P* = 0.0004). Trabecular thickness (Tb.Th) *n* = 12 studies showed overall significant increase in Sr-coated group (21.11 mm^−3^, *P* < 0.00001), trabecular number (Tb.N) *n* = 8 as will increased significantly (27.64 (1/mm), *P* < 0.00001), and trabecular spacing (Tb.Sp) *n* = 9 studies significantly favored Sr-coated group (−151.02 mm^−3^, *P* < 0.00001). Considerable heterogeneity was observed in all the present secondary analyses (I² ranged from 88% to 100%). To sum up, the analyzed studies displayed higher BV/TV, Conn.D, Tb. N, and Tb.Th, along with reduced Tb.Sp in the Sr-coated group.

### Biomechanical testing

**Fig. 6:**
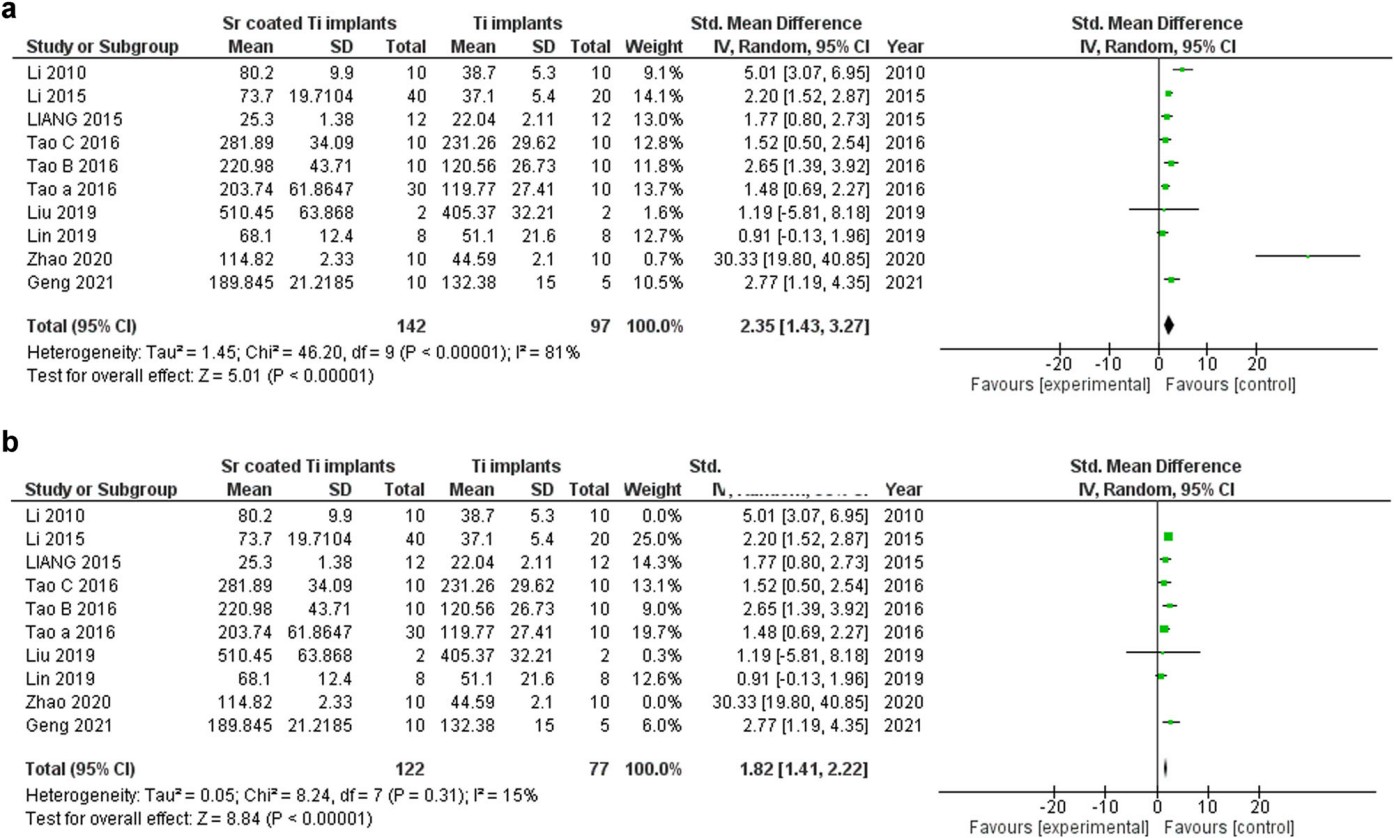
Biomechanical tests forest plots senstivity analysis. **a** Biomechanical tests forest plots. **b** Biomechanical tests forest plots after excluding the sources of heterogenicity and performing sub-group analysis.

The meta-synthesis of biomechanical tests included 10 studies of which four studies used maximum push-out test, three studies used maximum pull-out test, two studies used removal torque and one study used maximum push-in test. The overall SMD is 2.05 (*P* < 0.00001) which indicates a large effect in favor of Sr-coated implants according to Cohen’s rules of thumb [45], however the overall analysis resulted in moderate heterogeneity ((*P* = 0.02); I² = 56%). The sensitivity analysis showed that two studies were responsible for the heterogeneity, excluding both studies resulted in homogenous results in favor of Sr-coated implants (*P* = 0.31); I² = 15%) (Fig. 6).

## Discussion

Continuous advancements in dental implant material, design, surface treatment, as well as developments in surgical techniques, have not only shortened treatment time but also expanded the indications for implant therapy where a larger population of patients are now candidates for implant therapy. As a result, there is a growing interest in patients who have disease-related factors that may impact implant integration and success.

Osteoporosis is a systemic bone metabolic disease that affect implant osseointegration and therefore is considered as a potential risk factors for implant failure [46, 47]. Hence, the development of new therapeutic approaches should consider these physiologic determinants. The included studies compared the osseointegration of Sr coated titanium implants in simulated osteoporotic animal models versus Sr free implants.

Findings of this systematic review demonstrate the catalytic influence of Sr-modified implant surfaces in implant osseointegration in osteoporotic conditions based on the results of histomorphometric, microcomputed, and biomechanical analysis for the implant-bone samples. Moreover, the meta-analysis of the primary outcomes (BIC, biomechanical tests) supports the significant increase in the percentage of BIC and biomechanical test values in favor of Sr-modified implant surfaces.

This systematic review aimed to investigate the effect of Sr-modified implant surfaces on enhancing osseointegration and bone apposition in animals with metabolic osteoporotic conditions, in addition, a meta-analysis was performed to quantify the potential effect of Sr-coated surfaces on peri-implant bone apposition in terms of BIC, biomechanical integration and trabecular bone architecture.

Recently wide range of strontium compounds emerged in treating bone defects through systemic administration of drugs such as Sr ranelate or direct delivery of local agents such as injectable Sr-HA containing bioactive bone cement and Sr-doped ceramics to accelerate the bone healing process. However, Sr delivered systemically does not reach high enough concentrations inside the bone to produce a significant biological stimulation of bone formation [48]. Moreover, it poses serious side effects as increased incidence of venous thromboembolism (VTE), non-fatal myocardial infarction [49] and recently it has been contraindicated in patients with uncontrolled hypertension, ischemic heart disease, peripheral arterial disease, and/or cerebrovascular disease [50]. On the other hand, local delivery of strontium compounds resulted in enhanced bone growth and osseointegration at the bone-implant interface [42, 51]. Furthermore, Offermanns et al. have utilized the Atomic Absorption Spectrometry (AAS) to measure the serum levels of strontium to address any potential systemic effect of the released strontium from the titanium surface [18]. These authors reported that spectrometry measurements did not indicate any potential systemic effect by the local release of strontium from the implant surface [18]. Thus, the main concern to boost implant bone integration should be directed towards Sr-modified implant surfaces, especially in cases where bone quality is questionable.

Interestingly, all the included studies attributed the enhanced implant osseointegration and bone architecture to the local leaching of Sr ions. Strontium has a dual action aims to overcome the accelerated bone loss associated with osteoporosis by rebalancing bone remodeling in favor of bone formation through a calcium-sensing receptor (CaR)-mediated mechanism, on one hand Sr increases bone apposition by promoting pre-osteoblastic cell differentiation and inducing osteoblast survival and proliferation through the Canonical Wnt Signaling (Wnt/β-Catenin Pathway) [52], on the other hand, Sr directly induces osteoclast apoptosis through a signaling pathway dependent on the activation of diacylglycerol (DAG)-protein kinase C (PKC) βII [53], and indirectly reduces osteoclast development and activity by increasing the expression of osteoprotegerin (OPG) and decreasing the expression of receptor activator of nuclear factor kB ligand (RANKL) [54]. In addition, Sr coated implants have increased surface roughness that promotes osteoinductive cells attachment to the implant surface which works in tandem with the released Sr ions to improve implant osseointegration [21].

In the current study, consistent results were obtained from the histomorphometric, biomechanical and micro-CT evaluation techniques. All the analyzed osseointegration parameters showed positive results in favor of Sr-incorporated implants. Such consistency in results were in agreement with those of previous reviews that address the effect of Sr coated titanium implants on the osseointegration in healthy, non-osteoporotic conditions [20, 21]. and were in agreement with previous preclinical animal studies of Sr- incorporated bioactive glass scaffolds and bone cement containing biomaterials [55, 56].

In this study, the primary outcome variables were the BIC% and bone area (BA) recorded by the histomorphometric analysis, and the biomechanical tests. Results of the histomorphometrical assessment were prioritized over microcomputed tomography (µCT) when both approaches were used to assess the same outcome, and this preference was in agreement with Zhou et al. and Zhu Y et al. [44, 57], as it provides the most precise method of recording morphological changes at the implant-bone interface [58], and it is considered the most accurate way to assess implant osseointegration [59]. All the included studies that investigated BIC% reported significant results in favor of Sr-coating, and this was solidified by the meta-analysis. Moreover, the bone area (BA) was analyzed to evaluate the quality and quantity of peri-implant bone apposition as it reflects the new mineralized bone tissue area inside all implant threads and plays an important role in assessing the osteoconductive property of biomaterials. Eight included studies have evaluated the bone area, all of them reported significant improvement for Sr modified implant surfaces compared to Sr free implant surfaces. In addition, biomechanical tests were analyzed, as these tests are sensitive to changes in the mechanical properties of the bone-implant interface to predict its stability against various applied forces as healing progress [59]. Sr coated implants showed a significant increase in implant fixation compared to Sr-free implants.

For the secondary outcomes, the micro-computed tomography analysis was performed as it provide a nondestructive and a comprehensive evaluation of the trabecular architecture of peri-implant bone tissue [60]. Bone microstructure parameters (BV/TV, Tb.Th, Tb.N, Tb.Sp and Conn.D) were analyzed to evaluate the quality and quantity of peri-implant bone apposition, and all the included studies showed significant bone architecture enhancement in favor of St-coated implants.

The selected studies in this systematic review have reported using various surface coating methods to incorporate Sr ions on implant surface; six studies used electrochemical deposition, four studies used magnetron sputtering, three studies used hydrothermal treatment, three studies used micro-arc oxidation (MAO), two studies used sol-gel technique, and one study used chemical coprecipitation, however coating methods didn’t have any significant influence in the BIC%. Such results were consistent with findings obtained by López-Valverde et al. [21].

The present meta-analysis shows considerable heterogeneity, this disparity could be due to the different surface topography, Sr concentration in titanium implant surfaces, the different methods for incorporating Sr into implant surfaces, and follow-up periods, furthermore, subgroup analysis based on animal type or implant location did not manage to explain the heterogeneity. In addition, the variations in osteoporotic models resulted from the time between ovariectomy and experimental procedure where most of the studies inserted the implants after 8–14 weeks for rats and rabbits and 12 months for sheep, and one study inserted the implants only after 5 weeks after osteoporosis induction by OVX surgery in rat model, those time points represent diverse stages of osteoporosis; hence, these comparisons are heterogeneous. Furthermore, only eight studies confirmed the successful establishment of the osteoporotic condition.

The current systematic review included several limitations. Firstly, only one study evaluated implants placed in the jawbones, while the remaining studies used the tibia and/or femur. Secondly, although several studies have reported significant enhancement in bone architecture and implant osseointegration when Sr concentrations were increased from 0% Sr to 100% Sr in the implant surface [36, 37], many studies neglected to mention the Sr concentration in the intervention groups. So, we couldn’t conclude the ideal Sr concentration that should be used to enhance the osseointegration. Thirdly, none of the implants in the included studies were loaded. Therefore, future studies should be performed to evaluate the effect of Sr-coated implants under loading conditions, and to identify the ideal coating methods for incorporating Sr into the implant surface, and the ideal Sr concentrations. Furthermore, clinical evaluations based on well-designed randomized controlled clinical trials are required to address the effect of strontium coating of titanium implants in enhancing the osseointegration in patients with osteoporotic conditions.

## Conclusion

In summary, the present results provide evidence that strontium-coated titanium implants enhanced the osseointegration in animal models under osteoporotic condition as this surface modification techniques have improved the mechanical and biological properties of the titanium implants.

## Supplementary information

Supplementary Information

## Data Availability

All generated data in this research are available upon request.

## References

[1] Buser D, Janner SF, Wittneben JG, Brägger U, Ramseier CA, Salvi GE. 10‐year survival and success rates of 511 titanium implants with a sandblasted and acid‐etched surface: a retrospective study in 303 partially edentulous patients. Clin. Implant. Dent. Relat. Res. 2012;14:839–51. 22897683 10.1111/j.1708-8208.2012.00456.x

[2] Albrektsson T, Brånemark P-I, Hansson H-A, Lindström J. Osseointegrated titanium implants: requirements for ensuring a long-lasting, direct bone-to-implant anchorage in man. Acta Orthopaedica Scandinavica. 1981;52:155–70. 7246093 10.3109/17453678108991776

[3] Hwang D, Wang H-L. Medical contraindications to implant therapy: Part II: Relative contraindications. Implant. Dent. 2007;16:13–23. 17356368 10.1097/ID.0b013e31803276c8

[4] Al-Mahalawy H, Marei HF, Abuohashish H, Alhawaj H, Alrefaee M, Al-Jandan B. Effects of cisplatin chemotherapy on the osseointegration of titanium implants. J. Cranio-Maxillofacial Surg. 2016;44:337–46. 10.1016/j.jcms.2016.01.012 26895777

[5] Al-Jandan B, Marei HF, Abuohashish H, Zakaria O, Al-Mahalawy H. Effects of sunitinib targeted chemotherapy on the osseointegration of titanium implants. Biomedicine Pharmacotherapy. 2018;100:433–40. 29471246 10.1016/j.biopha.2018.02.056

[6] Raisz LG. Pathogenesis of osteoporosis: concepts, conflicts, and prospects. J. Clin. investigation. 2005;115:3318–25. 10.1172/JCI27071 PMC1297264 16322775

[7] Chen C-H, Wang L, Tulu US, Arioka M, Moghim MM, Salmon B, et al. An osteopenic/osteoporotic phenotype delays alveolar bone repair. Bone. 2018;112:212–9. 29704698 10.1016/j.bone.2018.04.019 PMC13561117

[8] Cho P, Schneider G, Krizan K, Keller J. Examination of the bone–implant interface in experimentally induced osteoporotic bone. Implant. Dent. 2004;13:79–87. 15017309 10.1097/01.id.0000116456.76235.bc

[9] Yıldız A, Esen E, Kürkçü M, Damlar İ, Dağlıoğlu K, Akova T. Effect of zoledronic acid on osseointegration of titanium implants: an experimental study in an ovariectomized rabbit model. J. Oral. Maxillofac. Surg. 2010;68:515–23. 20171470 10.1016/j.joms.2009.07.066

[10] ZAMAI RS, CORRÊA MG, RIBEIRO FV, CIRANO FR, CASATI MZ, MESSORA MR, et al. Does resveratrol favor peri-implant bone repair in rats with ovariectomy-induced osteoporosis? Gene expression, counter-torque micro-CT analysis. Braz. Oral. Res. 2023;37:e003. 36700588 10.1590/1807-3107bor-2023.vol37.0003

[11] Basudan AM, Shaheen MY, de Vries RB, van den Beucken JJ, Jansen JA, Alghamdi HS. Antiosteoporotic drugs to promote bone regeneration related to titanium implants: a systematic review and meta-analysis. Tissue Eng. Part. B: Rev. 2019;25:89–99. 30191772 10.1089/ten.TEB.2018.0120

[12] Park J-W, Seo J-H, Lee H-J. Enhanced osteogenic differentiation of mesenchymal stem cells by surface lithium modification in a sandblasted/acid-etched titanium implant. J. Biomater. Appl. 2022;37:447–58. 35594165 10.1177/08853282221104242

[13] Kim H, Choi S-H, Ryu J-J, Koh S-Y, Park J-H, Lee I-S. The biocompatibility of SLA-treated titanium implants. Biomed. Mater. 2008;3:025011. 18458368 10.1088/1748-6041/3/2/025011

[14] Deeks ED, Dhillon S. Strontium ranelate: a review of its use in the treatment of postmenopausal osteoporosis. Drugs. 2010;70:733–59. 20394457 10.2165/10481900-000000000-00000

[15] Saidak Z, Marie PJ. Strontium signaling: molecular mechanisms and therapeutic implications in osteoporosis. Pharmacology therapeutics. 2012;136:216–26. 22820094 10.1016/j.pharmthera.2012.07.009

[16] Peng S, Liu XS, Huang S, Li Z, Pan H, Zhen W, et al. The cross-talk between osteoclasts and osteoblasts in response to strontium treatment: involvement of osteoprotegerin. Bone. 2011;49:1290–8. 21925296 10.1016/j.bone.2011.08.031

[17] Li Y, Fu Q, Qi Y, Shen M, Niu Q, Hu K, et al. Effect of a hierarchical hybrid micro/nanorough strontium-loaded surface on osseointegration in osteoporosis. RSC Adv. 2015;5:52296–306.

[18] Offermanns V, Andersen OZ, Riede G, Sillassen M, Jeppesen CS, Almtoft KP, et al. Effect of strontium surface-functionalized implants on early and late osseointegration: A histological, spectrometric and tomographic evaluation. Acta Biomaterialia. 2018;69:385–94. 29425718 10.1016/j.actbio.2018.01.049

[19] Lin G, Zhou C, Lin M, Xu A, He F. Strontium‐incorporated titanium implant surface treated by hydrothermal reactions promotes early bone osseointegration in osteoporotic rabbits. Clin. oral. Implant. Res. 2019;30:777–90. 10.1111/clr.13460 31104360

[20] Shi J, Li Y, Gu Y, Qiao S, Zhang X, Lai H. Effect of titanium implants with strontium incorporation on bone apposition in animal models: A systematic review and meta-analysis. Sci. Rep. 2017;7:1–10. 29138499 10.1038/s41598-017-15488-1 PMC5686172

[21] López-Valverde N, Muriel-Fernández J, Gómez de Diego R, Ramírez JM, López-Valverde A. Effect of Strontium-Coated Titanium Implants on Osseointegration in Animal Models: A Literature Systematic Review. Int. J. Oral. Maxillofac. Implant. 2019;34:1389–96. 10.11607/jomi.7827 31711080

[22] Lu W, Zhou Y, Yang H, Cheng Z, He FJTJOPD. Efficacy of strontium supplementation on implant osseointegration under osteoporotic conditions: A systematic review. J. Prosthet. Dent. 2022;128:341–9. 33589234 10.1016/j.prosdent.2020.12.031

[23] Page MJ, Moher D, Bossuyt PM, Boutron I, Hoffmann TC, Mulrow CD, et al. PRISMA 2020 explanation and elaboration: updated guidance and exemplars for reporting systematic reviews. bmj. 2021;372:n160. 33781993 10.1136/bmj.n160 PMC8005925

[24] Higgins JP, Thomas J, Chandler J, Cumpston M, Li T, Page MJ, et al. Cochrane handbook for systematic reviews of interventions: 2nd Edition. Chichester (UK): John Wiley & Sons; 2019.

[25] Hooijmans CR, Rovers MM, De Vries RB, Leenaars M, Ritskes-Hoitinga M, Langendam MW. SYRCLE’s risk of bias tool for animal studies. BMC Med. Res. Methodol. 2014;14:1–9. 24667063 10.1186/1471-2288-14-43 PMC4230647

[26] Kilkenny C, Browne WJ, Cuthill IC, Emerson M, Altman DG. Improving bioscience research reporting: the ARRIVE guidelines for reporting animal research. J. Pharmacology Pharmacotherapeutics. 2010;1:94–9. 10.4103/0976-500X.72351 PMC3043335 21350617

[27] Geng Z, Ji L, Li Z, Wang J, He H, Cui Z, et al. Nano-needle strontium-substituted apatite coating enhances osteoporotic osseointegration through promoting osteogenesis and inhibiting osteoclastogenesis. Bioact. Mater. 2020;6:905–15. 10.1016/j.bioactmat.2020.09.024 PMC7553892 33102935

[28] Katunar MR, Pastore JI, Cisilino A, Merlo J, Alonso LS, Baca M, et al. Early osseointegration of strontium-doped coatings on titanium implants in an osteoporotic rat model. Surf. Coat. Technol. 2022;433:128159.

[29] Li Y, Li Q, Zhu S, Luo E, Li J, Feng G, et al. The effect of strontium-substituted hydroxyapatite coating on implant fixation in ovariectomized rats. Biomaterials. 2010;31:9006–14. 20800275 10.1016/j.biomaterials.2010.07.112

[30] Li Y, Luo E, Zhu S, Li J, Zhang L, Hu JJJOAB, et al. Cancellous bone response to strontium-doped hydroxyapatite in osteoporotic rats. J. Appl. Biomater. Funct. Mater. 2015;13:28–34. 24744229 10.5301/jabfm.5000168

[31] Liang Y, Li H, Xu J, Li X, Li X, Yan Y, et al. Strontium coating by electrochemical deposition improves implant osseointegration in osteopenic models. Exp. therapeutic Med. 2015;9:172–6. 10.3892/etm.2014.2038 PMC4247308 25452797

[32] Lin G, Zhou C, Lin M, Xu A, He FJ. Strontium‐incorporated titanium implant surface treated by hydrothermal reactions promotes early bone osseointegration in osteoporotic rabbits. Clin. Oral. Implant. Res. 2019;30:777–90. 10.1111/clr.13460 31104360

[33] Liu F, Li Y, Liang J, Sui W, Bellare A, Kong LJCID, et al. Effects of micro/nano strontium‐loaded surface implants on osseointegration in ovariectomized sheep. Clin. Implant. Dent. Relat. Res. 2019;21:377–85. 30715786 10.1111/cid.12719

[34] Mi B, Xiong W, Xu N, Guan H, Fang Z, Liao H, et al. Strontium-loaded titania nanotube arrays repress osteoclast differentiation through multiple signalling pathways: In vitro and in vivo studies. Sci. Rep. 2017;7:2328. 28539667 10.1038/s41598-017-02491-9 PMC5443803

[35] Offermanns V, Andersen OZ, Riede G, Andersen IH, Almtoft KP, Sørensen S, et al. Bone regenerating effect of surface-functionalized titanium implants with sustained-release characteristics of strontium in ovariectomized rats. Int. J. Nanomed. 2016;11:2431–42. 10.2147/IJN.S101673 PMC4892864 27313456

[36] Shen X, Fang K, Yie KHR, Zhou Z, Shen Y, Wu S, et al. High proportion strontium-doped micro-arc oxidation coatings enhance early osseointegration of titanium in osteoporosis by anti-oxidative stress pathway. Bioact. Mater. 2022;10:405–19. 34901556 10.1016/j.bioactmat.2021.08.031 PMC8636681

[37] Tao Z-S, Bai B-L, He X-W, Liu W, Li H, Zhou Q, et al. A comparative study of strontium-substituted hydroxyapatite coating on implant’s osseointegration for osteopenic rats. Med. Biol. Eng. Comput. 2016;54:1959–68. 27099156 10.1007/s11517-016-1494-9

[38] Tao Z-S, Zhou W-S, He X-W, Liu W, Bai B-L, Zhou Q, et al. A comparative study of zinc, magnesium, strontium-incorporated hydroxyapatite-coated titanium implants for osseointegration of osteopenic rats. Mater Sci. Eng. C. Mater. Biol. Appl. 2016;62:226–32. 26952418 10.1016/j.msec.2016.01.034

[39] Tao Z-S, Zhou W-S, Qiang Z, Tu K-k, Huang Z-L, Xu H-M, et al. Intermittent administration of human parathyroid hormone (1–34) increases fixation of strontium-doped hydroxyapatite coating titanium implants via electrochemical deposition in ovariectomized rat femur. J. Biomater. Appl. 2016;30:952–60. 26482573 10.1177/0885328215610898

[40] Wang H, Xu Q, Hu H, Shi C, Lin Z, Jiang H, et al. The fabrication and function of strontium-modified hierarchical micro/nano titanium implant. Int. J. Nanomed. 2020;15:8983–98. 10.2147/IJN.S268657 PMC7682802 33239873

[41] Wen J, Li J, Pan H, Zhang W, Zeng D, Xu L, et al. Strontium delivery on topographical titanium to enhance bioactivity and osseointegration in osteoporotic rats. J. Mater. Chem. B. 2015;3:4790–804. 32262668 10.1039/c5tb00128e

[42] Zhang J, Liu L, Zhao S, Wang H, Yang G. Characterization and In Vivo Evaluation of Trace Element-Loaded Implant Surfaces in Ovariectomized Rats. Int. J. Oral. Maxillofac. Implant. 2015;30:1105–12. 10.11607/jomi.3906 26394347

[43] Zhao B, Li X, Xu H, Jiang Y, Wang D, Liu R. Influence of simvastatin-strontium-hydroxyapatite coated implant formed by micro-arc oxidation and immersion method on osteointegration in osteoporotic rabbits. Int. J. Nanomed. 2020;15:1797–807. 10.2147/IJN.S244815 PMC7083628 32214812

[44] Zhu Y, Zheng T, Wen L-M, Li R, Zhang Y-B, Bi W-J, et al. Osteogenic capability of strontium and icariin-loaded TiO2 nanotube coatings in vitro and in osteoporotic rats. J. Biomater. Appl. 2021;35:1119–31. 33632004 10.1177/0885328221997998

[45] Cohen J. Statistical power analysis. Curr. directions psychological Sci. 1992;1:98–101.

[46] Mombelli A, Cionca N. Systemic diseases affecting osseointegration therapy. Clin. oral. Implant. Res. 2006;17:97–103. 10.1111/j.1600-0501.2006.01354.x 16968386

[47] Ikebe K, Wada M, Kagawa R, Maeda Y. Is old age a risk factor for dental implants? Japanese Dental Sci. Rev. 2009;45:59–64.

[48] Marx D, Yazdi AR, Papini M, Towler M. A review of the latest insights into the mechanism of action of strontium in bone. Bone Rep. 2020;12:100273. 32395571 10.1016/j.bonr.2020.100273 PMC7210412

[49] Abrahamsen B, Grove E, Vestergaard P. Nationwide registry-based analysis of cardiovascular risk factors and adverse outcomes in patients treated with strontium ranelate. Osteoporos. Int. 2014;25:757–62. 24322475 10.1007/s00198-013-2469-4

[50] Agenc EM Strontium ranelate. Summary of product characteristics. European Medicines Agency. Available at: https://ec.europa.eu/health/documents/communityregister/2014/20140522128592/anx_128592_en.pdf (Accessed: 2024).

[51] Liu W, Wang T, Yang C, Darvell B, Wu J, Lin K, et al. Alkaline biodegradable implants for osteoporotic bone defects—importance of microenvironment pH. Osteoporos. Int. 2016;27:93–104. 26134681 10.1007/s00198-015-3217-8

[52] Rybchyn MS, Slater M, Conigrave AD, Mason RS. An Akt-dependent increase in canonical Wnt signaling and a decrease in sclerostin protein levels are involved in strontium ranelate-induced osteogenic effects in human osteoblasts. J. Biol. Chem. 2011;286:23771–9. 21566129 10.1074/jbc.M111.251116 PMC3129158

[53] Hurtel-Lemaire AS, Mentaverri R, Caudrillier A, Cournarie F, Wattel A, Kamel S, et al. The calcium-sensing receptor is involved in strontium ranelate-induced osteoclast apoptosis: new insights into the associated signaling pathways. J. Biol. Chem. 2009;284:575–84. 18927086 10.1074/jbc.M801668200

[54] Brennan TC, Rybchyn MS, Green W, Atwa S, Conigrave AD, Mason RS. Osteoblasts play key roles in the mechanisms of action of strontium ranelate. Br. J. pharmacology. 2009;157:1291–300. 10.1111/j.1476-5381.2009.00305.x PMC2743848 19563530

[55] Wu CC, Kuo CL, Fan FY, Yang KC. Strontium‐impregnated bioabsorbable composite for osteoporotic fracture fixation. J. Biomed. Mater. Res. Part. A. 2015;103:3355–63. 10.1002/jbm.a.35471 25847487

[56] Wei L, Ke J, Prasadam I, Miron RJ, Lin S, Xiao Y, et al. A comparative study of Sr-incorporated mesoporous bioactive glass scaffolds for regeneration of osteopenic bone defects. Osteoporos. Int. 2014;25:2089–96. 24807629 10.1007/s00198-014-2735-0

[57] Zhou C, Chen Y, Zhu Y, Lin G, Zhang L, Liu X, et al. Antiadipogenesis and osseointegration of strontium-doped implant surfaces. J. dental Res. 2019;98:795–802. 10.1177/0022034519850574 31136719

[58] Meredith N, Shagaldi F, Alleyne D, Sennerby L, Cawley P. The application of resonance frequency measurements to study the stability of titanium implants during healing in the rabbit tibia. Clin. oral. Implant. Res. 1997;8:234–43. 10.1034/j.1600-0501.1997.080310.x 9586468

[59] Seong W-J, Grami S, Jeong SC, Conrad HJ, Hodges JS. Comparison of Push-In versus Pull-Out Tests on Bone-Implant Interfaces of Rabbit Tibia Dental Implant Healing Model. Clin. Implant. Dent. Relat. Res. 2013;15:460–9. 22172015 10.1111/j.1708-8208.2011.00357.x

[60] Rebaudi A, Koller B, Laib A, Trisi P. Microcomputed tomographic analysis of the peri-implant bone. Int. J. Periodontics Restor. Dent. 2004;24:316–25. 15446401

