# Supplementary material for: Does strontium coated titanium implants enhance the osseointegration in animal models under osteoporotic condition? A systematic review and meta-analysis
Source: BDJ Open. 2024 Aug 24;10:69. doi: 10.1038/s41405-024-00220-9 (PMC11344846; doi:10.1038/s41405-024-00220-9)
Supplement: Supplementary file 1 — Supplementary Information [file 41405_2024_220_MOESM1_ESM.pdf]

The detailed and comprehensive research strategy:

A comprehensive search was conducted, including the databases MEDLINE via PubMed, Web of Science, Scopus, and Ebsco, without language restrictions. The following keywords were used as search terms in PubMed: [("strontium" OR "Sr" OR "Strontium[Mesh] OR Strontium-incorporated OR Strontium-surface OR strontium-coat OR strontium-coating OR strontium-oxide OR Sr-coat OR Sr-HA OR NT-Sr OR nano-Sr OR nano-strontium OR strontium-substituted OR strontium-functionalized OR strontium-loaded OR Strontium-modified OR Sr-modification OR SLA-Sr) AND (titanium OR Ti OR implant OR implantation OR implants OR SLA OR "Titanium[Mesh] OR "Dental Implants[Mesh]) AND ("osteoporosis OR osteoporotic OR osteopenia OR osteopenic)].

Plus, the database-specific limiters:

For Web of Science:

("strontium" OR "Sr" OR Strontium-incorporated OR Strontium-surface OR strontium-coat OR strontium-coating OR strontium-oxide OR Sr-coat OR Sr-HA OR NT-Sr OR nano-Sr OR nano-strontium OR strontium-substituted OR strontium-functionalized OR strontium-loaded OR Strontium-modified OR Sr-modification OR SLA-Sr) AND (titanium OR Ti OR implant OR implantation OR implants OR SLA OR Dental Implants) AND (osteoporosis OR osteoporotic OR osteopenia OR osteopenic)

For Scopus:

strontium OR sr OR "strontium incorporated" OR "strontium surface" OR "strontium coat" OR "strontium coating" OR "strontium oxide" OR "sr coat" OR "sr-HA" OR "NT-sr" OR "nano sr" OR "nano strontium" OR "strontium substituted" OR "strontium functionalized" OR "strontium loaded" OR "strontium modified" OR "sr modification" OR "SLA-Sr"

titanium OR "ti" OR implant OR implantation OR implants OR "SLA" OR "dental implants"

"osteoporosis" OR osteoporotic OR osteopenia OR osteogenic

For EBSCO:

(strontium OR sr OR "strontium incorporated" OR "strontium surface" OR "strontium coat" OR "strontium coating" OR "strontium oxide" OR "sr coat" OR "sr-HA" OR "NT-sr" OR "nano sr" OR

"nano strontium " OR "strontium substituted" OR "strontium functionalized" OR "strontium loaded" OR "strontium modified" OR "sr modification" OR "SLA-Sr") AND (titanium OR "ti" OR implant OR implantation OR implants OR "SLA" OR "dental implants") AND ("osteoporosis" OR osteoporotic OR osteopenia OR osteogenic)

Furthermore, clinical trial registries for ongoing or recently completed trials were searched.

Full Text Excluded Studies

Year	authors	Title	Justification
2013	C. E. Cardemil, I.;Xia, W.;Emanuelsson, L.;Norlindh, B.;Omar, O.;Thomsen, P.	Strontium-Doped Calcium Phosphate and Hydroxyapatite Granules Promote Different Inflammatory and Bone Remodelling Responses in Normal and Ovariectomised Rats	No Ti implants
2016	S. B. Chandran, S. S.;Krishnan, V. S. H.;Varma, H. K.;John, A.	Osteogenic efficacy of strontium hydroxyapatite micro-granules in osteoporotic rat model	No Ti implants
2013	B. L. Chen, Y.;Yang, X.;Xu, H.;Xie, D.	Zoledronic acid enhances bone-implant osseointegration more than alendronate and strontium ranelate in ovariectomized rats	No Sr coating for Ti implants
2016	Z. B. X. Du, Y.;Hashimi, S.;Hamlet, S. M.;Ivanovski, S.	The effects of implant topography on osseointegration under estrogen deficiency induced osteoporotic conditions: Histomorphometric, transcriptional and ultrastructural analysis	No Sr coating for Ti implants
2013	G. X. Hulsart-Billström, W.;Pankotai, E.;Weszl, M.;Carlsson, E.;Forster-Horváth, C.;Larsson, S.;Engqvist, H.;Lacza, Z.	Osteogenic potential of Sr-doped calcium phosphate hollow spheres in vitro and in vivo	No Ti implants
2013	S. O. Jebahi, H.;Elleuch, J.;Tounsi, S.;Keskes, H.;pellen, P.;Rebai, T.;El Feki, A.;El Feki, H.	The potential restorative effects of strontium-doped bioactive glass on bone microarchitecture after estrogen-deficieny induced osteoporosis: Physicochemical and histomorphometric analyses	No Ti implants
2021	C. T. C. Kao, Y. C.;Lee, A. K. X.;Lin, Y. H.;Huang, T. H.;Liu, Y. C.;Shie, M. Y.	The synergistic effects of Xu Duan combined Sr-contained calcium silicate/poly-ε-caprolactone scaffolds for the promotion of osteogenesis marker expression and the induction of bone regeneration in osteoporosis	No Ti implants
2010	Y. F. Li, G.;Gao, Y.;Luo, E.;Liu, X.;Hu, J.	Strontium ranelate treatment enhances hydroxyapatite-coated titanium screws fixation in osteoporotic rats	No Sr coating for Ti implants
2012	Y. L. Li, X.;Song, G.;Chen, K.;Yin, G.;Hu, J.	Effects of strontium ranelate on osseointegration of titanium implant in osteoporotic rats	No Sr coating for Ti implants
2017	Y. F. Okuzu, S.;Yamaguchi, S.;Yamamoto, K.;Shimizu, T.;Sono, T.;Goto, K.;Otsuki, B.;Matsushita, T.;Kokubo, T.;Matsuda, S.	Strontium and magnesium ions released from bioactive titanium metal promote early bone bonding in a rabbit implant model	non-osteoporotic animal models

2017	M. P. Rohnke, S.;Mogwitz, B.;Henß, A.;Thomas, J.;Bieberstein, D.;Gemming, T.;Otto, S. K.;Ray, S.;Schumacher, M.;Gelinsky, M.;Alt, V.	Strontium release from Sr2+-loaded bone cements and dispersion in healthy and osteoporotic rat bone	No Ti implants
2021	Y. L. Sun, Y.;Zhang, Y.;Wang, T.;Lin, K.;Liu, J.	A polydopamine-assisted strontium-substituted apatite coating for titanium promotes osteogenesis and angiogenesis via FAK/MAPK and PI3K/AKT signaling pathways	non-osteoporotic animal models
2021	H. F. Wang, X.;Shi, J.;Li, L.;Sun, J.;Zhang, X.;Han, Q.;Deng, Y.;Gan, X.	Nutrient Element Decorated Polyetheretherketone Implants Steer Mitochondrial Dynamics for Boosted Diabetic Osseointegration	No Ti implants
2021	X. G. Wang, J.;Wen, J.;Zhang, X.;Cao, L.;Zeng, D.;Liu, X.;Jiang, X.	Novel vascular strategies on polyetheretherketone modification in promoting osseointegration in ovariectomized rats	No Ti implants
2014	L. K. Wei, J.;Prasadam, I.;Miron, R. J.;Lin, S.;Xiao, Y.;Chang, J.;Wu, C.;Zhang, Y.	A comparative study of Sr-incorporated mesoporous bioactive glass scaffolds for regeneration of osteopenic bone defects	No Ti implants
2015	C. C. K. Wu, C. L.;Fan, F. Y.;Yang, K. C.	Strontium-impregnated bioabsorbable composite for osteoporotic fracture fixation	No Ti implants
2021	Y. B. Z. Xu, L. F.;Xu, J. G.;Li, J.;Wang, H.;He, F. M.	Strontium-incorporated titanium implant surfaces treated by hydrothermal treatment enhance rapid osseointegration in diabetes: A preclinical vivo experimental study	non-osteoporotic animal models
2011	W. L. Z. Yang, Y.;Yang, J. H.;Tan, L. L.;Yang, K.	Potential antiosteoporosis effect of biodegradable magnesium implanted in STZ-induced diabetic rats	No Sr coated implants
2020	B. W. Yuan, L.;Zhao, R.;Yang, X.;Yang, X.;Zhu, X.;Liu, L.;Zhang, K.;Song, Y.;Zhang, X.	A biomimetically hierarchical polyetherketoneketone scaffold for osteoporotic bone repair	No Ti implants (used scaffolds)
2020	X. W. zhang, B.;Ma, L.;Xie, L.;Yang, H.;Li, Y.;Wang, S.;Qiao, H.;Lin, H.;Lan, J.;Huang, Y.	Chemical stability, antibacterial and osteogenic activities study of strontium-silver co-substituted fluorohydroxyapatite nanopillars: A potential multifunctional biological coating	non-osteoporotic animal models
2021	Z. C. J. Zhang, B.;Yang, H. T.;Han, Y.;Wu, Q.;Dai, K. R.;Zheng, Y. F.	Zn0.8Li0.1Sr-a biodegradable metal with high mechanical strength comparable to pure Ti for the treatment of osteoporotic bone fractures: In vitro and in vivo studies	Systemic drug adminstration

2021	D. W. Z. Zhao, K. Q.;Wang, K.;Sun, Z. Y.;Lu, Y. P.;Cheng, L.;Xiao, G. Y.;Liu, C.	Interleukin-4 assisted calcium-strontium-zinc-phosphate coating induces controllable macrophage polarization and promotes osseointegration on titanium implant	non-osteoporotic animal models
2019	C. C. Zhou, Y. Q.;Zhu, Y. H.;Lin, G. F.;Zhang, L. F.;Liu, X. C.;He, F. M.	Antiadipogenesis and Osseointegration of Strontium-Doped Implant Surfaces	non-osteoporotic animal models

Frost-Plot Subgroup analysis for bone-implant contact (BIC) according to the animal model, implantation location, and follow-up period

Study or Subgroup	Sr-coated Ti implants Mean	SD	Total	Ti implants Mean	SD	Total	Weight	Mean Difference IV, Random, 95% CI
**1.1.1 Pool analysis**								
Geng 2021	68.845	7.6053	10	38.86	4.38	5	2.0%	29.98 [23.91, 36.06]
Li 2015	55.59	8.71	10	33.99	5.35	10	2.0%	21.60 [15.26, 27.94]
Li 2010	63.64	10.26	10	42.37	7.87	10	1.9%	21.27 [13.26, 29.28]
Li 2015	41.88	11.2685	40	24.67	3.71	20	2.1%	17.21 [13.36, 21.06]
LIANG 2015	58.72	3.85	12	39.34	4.42	12	2.1%	19.38 [16.06, 22.70]
Lin 2019	75.8	6.9	8	68.7	6.8	8	2.0%	7.10 [0.39, 13.81]
Liu 2019	74.715	7.09	32	67.53	5.34	32	2.1%	7.19 [4.11, 10.26]
Offermanns 2016	82.0233	13.8335	21	81.26	8.66	7	1.9%	0.76 [-7.96, 9.49]
Tao a 2016	49.8933	16.7839	30	15.25	3.61	10	2.0%	34.64 [28.23, 41.05]
Tao b 2016	61.06	11.82	10	32.86	3.7	10	1.9%	28.20 [20.52, 35.88]
Tao c 2016	49.77	10.65	10	36.82	5.79	10	1.9%	12.95 [5.44, 20.46]
Wen 2015	56.99	6.83	8	24.18	7.75	8	2.0%	32.81 [25.65, 39.97]
Zhang 2015	58.6	2.9	6	51	6.6	6	2.0%	7.60 [1.83, 13.37]
Zhao 2020	71.45	2.17	7	34.48	1.12	7	2.1%	36.97 [35.16, 38.78]
Zhu 2021	42.69	2.8176	6	35.19	4.02	3	2.0%	7.50 [2.42, 12.58]
**Subtotal (95% CI)**			**220**			**158**	**29.9%**	**19.05 [12.02, 26.08]**
Heterogeneity: Tau² = 183.40; Chi² = 485.41, df = 14 (P < 0.00001); I² = 97%								
Test for overall effect: Z = 5.31 (P < 0.00001)								
**1.1.2 Rats**								
Li 2015	55.59	8.71	10	33.99	5.35	10	2.0%	21.60 [15.26, 27.94]
Li 2010	63.64	10.26	10	42.37	7.87	10	1.9%	21.27 [13.26, 29.28]
Li 2015	41.88	11.2685	40	24.67	3.71	20	2.1%	17.21 [13.36, 21.06]
LIANG 2015	58.72	3.85	12	39.34	4.42	12	2.1%	19.38 [16.06, 22.70]
Offermanns 2016	82.0233	13.8335	21	81.26	8.66	7	1.9%	0.76 [-7.96, 9.49]
Tao a 2016	49.8933	16.7839	30	15.25	3.61	10	2.0%	34.64 [28.23, 41.05]
Tao b 2016	61.06	11.82	10	32.86	3.7	10	1.9%	28.20 [20.52, 35.88]
Tao c 2016	49.77	10.65	10	36.82	5.79	10	1.9%	12.95 [5.44, 20.46]
Wen 2015	56.99	6.83	8	24.18	7.75	8	2.0%	32.81 [25.65, 39.97]
Zhang 2015	58.6	2.9	6	51	6.6	6	2.0%	7.60 [1.83, 13.37]
Zhu 2021	42.69	2.8176	6	35.19	4.02	3	2.0%	7.50 [2.42, 12.58]
**Subtotal (95% CI)**			**163**			**106**	**21.8%**	**18.54 [13.11, 23.97]**
Heterogeneity: Tau² = 73.51; Chi² = 97.45, df = 10 (P < 0.00001); I² = 90%								
Test for overall effect: Z = 6.69 (P < 0.00001)								
**1.1.3 Rabbits**								
Geng 2021	68.845	7.6053	10	38.86	4.38	5	2.0%	29.98 [23.91, 36.06]
Lin 2019	75.8	6.9	8	68.7	6.8	8	2.0%	7.10 [0.39, 13.81]
Zhao 2020	71.45	2.17	7	34.48	1.12	7	2.1%	36.97 [35.16, 38.78]
**Subtotal (95% CI)**			**25**			**20**	**6.1%**	**24.91 [8.21, 41.60]**
Heterogeneity: Tau² = 210.33; Chi² = 73.27, df = 2 (P < 0.00001); I² = 97%								
Test for overall effect: Z = 2.92 (P = 0.003)								
**1.1.4 sheeps**								
Liu 2019	74.715	7.09	32	67.53	5.34	32	2.1%	7.19 [4.11, 10.26]
**Subtotal (95% CI)**			**32**			**32**	**2.1%**	**7.19 [4.11, 10.26]**
Heterogeneity: Not applicable								
Test for overall effect: Z = 4.58 (P < 0.00001)								
**1.1.8 Tibia**								
Li 2010	63.64	10.26	10	42.37	7.87	10	1.9%	21.27 [13.26, 29.28]
LIANG 2015	58.72	3.85	12	39.34	4.42	12	2.1%	19.38 [16.06, 22.70]
Lin 2019	75.8	6.9	8	68.7	6.8	8	2.0%	7.10 [0.39, 13.81]
Offermanns 2016	82.0233	13.8335	21	81.26	8.66	7	1.9%	0.76 [-7.96, 9.49]
Zhang 2015	58.6	2.9	6	51	6.6	6	2.0%	7.60 [1.83, 13.37]
Zhao 2020	71.45	2.17	7	34.48	1.12	7	2.1%	36.97 [35.16, 38.78]
**Subtotal (95% CI)**			**64**			**50**	**12.0%**	**15.74 [3.12, 28.36]**
Heterogeneity: Tau² = 239.00; Chi² = 244.40, df = 5 (P < 0.00001); I² = 98%								
Test for overall effect: Z = 2.44 (P = 0.01)								
**1.1.9 femur**								
Geng 2021	68.845	7.6053	10	38.86	4.38	5	2.0%	29.98 [23.91, 36.06]
Li 2015	55.59	8.71	10	33.99	5.35	10	2.0%	21.60 [15.26, 27.94]
Li 2015	41.88	11.2685	40	24.67	3.71	20	2.1%	17.21 [13.36, 21.06]
Tao a 2016	49.8933	16.7839	30	15.25	3.61	10	2.0%	34.64 [28.23, 41.05]
Tao b 2016	61.06	11.82	10	32.86	3.7	10	1.9%	28.20 [20.52, 35.88]
Tao c 2016	49.77	10.65	10	36.82	5.79	10	1.9%	12.95 [5.44, 20.46]
Wen 2015	56.99	6.83	8	24.18	7.75	8	2.0%	32.81 [25.65, 39.97]
Zhu 2021	42.69	2.8176	6	35.19	4.02	3	2.0%	7.50 [2.42, 12.58]
**Subtotal (95% CI)**			**124**			**76**	**15.9%**	**22.98 [16.07, 29.89]**
Heterogeneity: Tau² = 88.98; Chi² = 74.95, df = 7 (P < 0.00001); I² = 91%								
Test for overall effect: Z = 6.52 (P < 0.00001)								
**1.1.10 mandible**								
Liu 2019	74.715	7.09	32	67.53	5.34	32	2.1%	7.19 [4.11, 10.26]
**Subtotal (95% CI)**			**32**			**32**	**2.1%**	**7.19 [4.11, 10.26]**
Heterogeneity: Not applicable								
Test for overall effect: Z = 4.58 (P < 0.00001)								
**1.1.11 short-follow up**								
Geng 2021	22.25	5.5634	10	9.53	1.69	5	2.1%	12.72 [8.97, 16.47]
LIANG 2015	58.72	3.85	12	39.34	4.42	12	2.1%	19.38 [16.06, 22.70]
Lin 2019	80.2	6.6	8	70.4	4.6	8	2.0%	9.80 [4.23, 15.37]
Zhang 2015	40.8	8.3	6	36.1	2.8	6	2.0%	4.70 [-2.31, 11.71]
Zhu 2021	44.465	2.1768	6	35.19	4.02	3	2.0%	9.28 [4.40, 14.15]
**Subtotal (95% CI)**			**42**			**34**	**10.2%**	**11.63 [6.68, 16.59]**
Heterogeneity: Tau² = 25.57; Chi² = 22.88, df = 4 (P = 0.0001); I² = 83%								
Test for overall effect: Z = 4.60 (P < 0.00001)								
**Total (95% CI)**			**702**			**508**	**100.0%**	**18.27 [14.72, 21.81]**
Heterogeneity: Tau² = 154.27; Chi² = 1574.14, df = 49 (P < 0.00001); I² = 97%								
Test for overall effect: Z = 10.10 (P < 0.00001)								
Test for subgroup differences: Chi² = 39.07, df = 7 (P < 0.00001), I² = 82.1%								

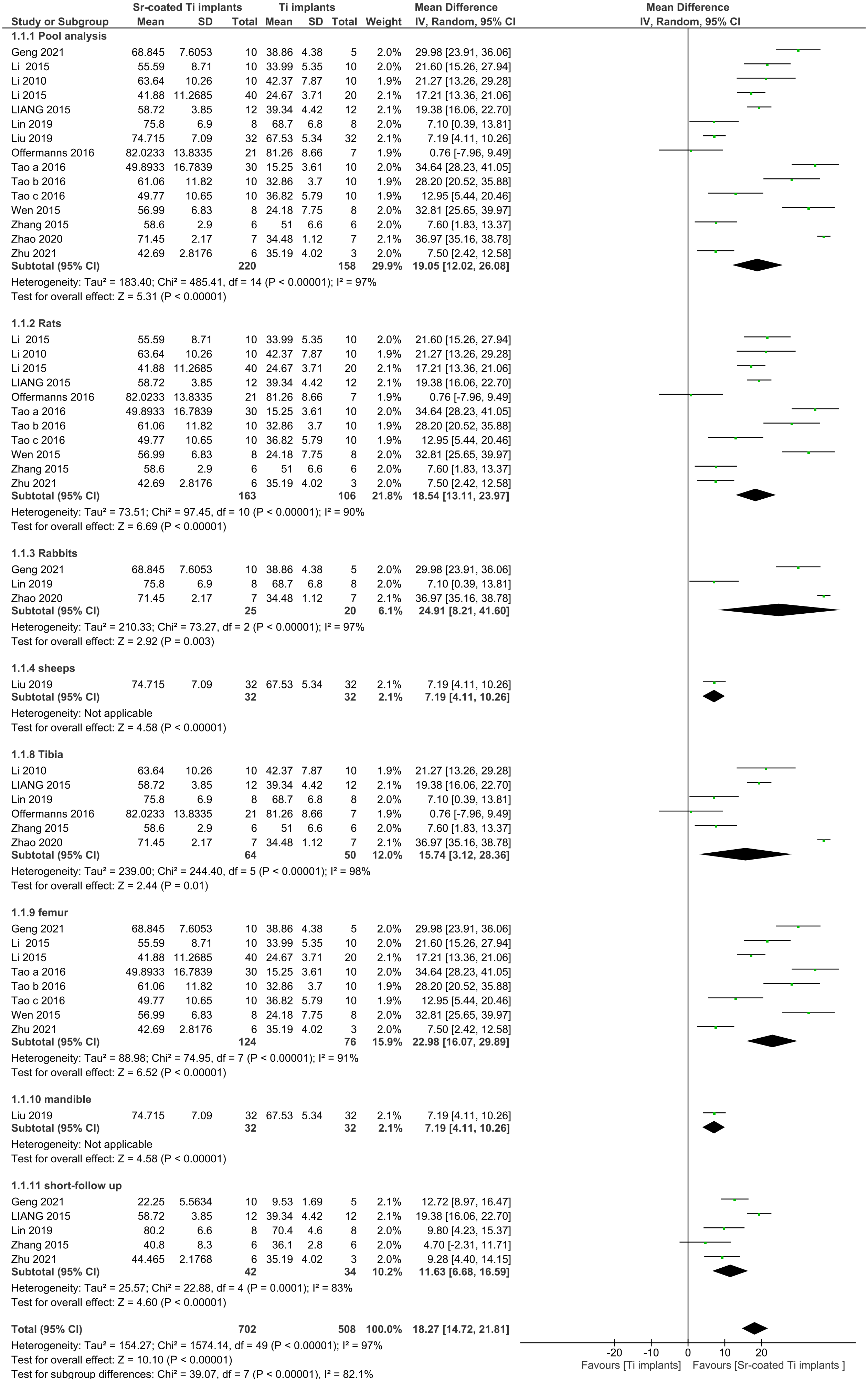

SYRCLE Risk of Bias

	1.Sequence generation	2.Baseline characteristics	3.Allocation concealment	4.Random housing	5.Blinding : caregivers and	6.Random outcome assessment	7.Blinding : outcome assessment	8.Incompelete outcome data	9.Selective outcome reporting	Other sources of bias
Geng 2021	unclear	low risk	unclear	unclear	unclear	high risk	unclear	low risk	low risk	unclear
Katunar 2022	unclear	low risk	unclear	unclear	unclear	low risk	unclear	unclear	low risk	unclear
Li 2015	low risk	low risk	unclear	unclear	unclear	unclear	unclear	unclear	low risk	unclear
Li 2010	low risk	low risk	unclear	low risk	unclear	high risk	unclear	high risk	low risk	unclear
Li 2015	low risk	low risk	unclear	low risk	unclear	low risk	unclear	low risk	low risk	unclear
Liang 2015	low risk	low risk	unclear	unclear	unclear	low risk	unclear	low risk	low risk	unclear
Lin 2019	low risk	low risk	unclear	low risk	unclear	high risk	unclear	low risk	low risk	unclear
Liu 2019	unclear	low risk	unclear	unclear	unclear	low risk	unclear	low risk	low risk	unclear
Mi 2017	unclear	low risk	unclear	low risk	unclear	low risk	unclear	low risk	low risk	unclear
Offermanns 2016	unclear	low risk	unclear	low risk	unclear	low risk	unclear	low risk	low risk	unclear
Shen 2022	low risk	unclear	unclear	unclear	unclear	low risk	unclear	low risk	low risk	unclear
Tao a 2016	low risk	low risk	unclear	low risk	unclear	low risk	unclear	low risk	low risk	unclear
Tao b 2016	low risk	low risk	unclear	low risk	unclear	low risk	unclear	low risk	low risk	unclear
Tao c 2016	low risk	low risk	unclear	low risk	unclear	low risk	unclear	low risk	low risk	unclear
Wang 2020	unclear	unclear	unclear	unclear	unclear	low risk	unclear	low risk	low risk	unclear
Wen 2015	low risk	low risk	unclear	unclear	unclear	low risk	unclear	low risk	low risk	unclear
Zhang 2015	low risk	low risk	unclear	unclear	unclear	low risk	unclear	low risk	low risk	unclear
Zhao 2020	low risk	low risk	unclear	unclear	unclear	low risk	unclear	low risk	low risk	unclear
Zhu 2021	unclear	low risk	unclear	low risk	unclear	low risk	unclear	low risk	low risk	unclear
